# Management of Anticoagulation for Portal Vein Thrombosis in Individuals with Cirrhosis: A Systematic Review

**DOI:** 10.1155/2012/672986

**Published:** 2012-06-20

**Authors:** Geneviève Huard, Marc Bilodeau

**Affiliations:** Liver Unit, Hôpital Saint-Luc, Centre Hospitalier de l'Université de Montréal, Montréal, QC, Canada H2X 3J4

## Abstract

Non-neoplastic portal vein thrombosis (PVT) is an increasingly recognized complication of liver cirrhosis. It is often diagnosed fortuitously and can be either partial or complete. The clinical significance of PVT is not obvious except in some situations such as when patients are on the waiting list for liver transplantation. The only known therapy is anticoagulation which has been shown to permit the disappearance of thrombosis and to prevent further extension. Anticoagulation is a challenging therapy in individuals with liver cirrhosis because of the well-recognized coagulation abnormalities observed in that setting and because of the increased risk of bleeding, especially from gastrointestinal tract caused by portal hypertension. We herein review the current knowledge on that topic in order to highlight the advantages and disadvantages of the currently proposed therapeutic attitudes in face of the diagnosis of PVT in individuals with cirrhosis.

## 1. Introduction 

Nonneoplastic portal vein thrombosis (PVT) is encountered in 0.6 to 26% of individuals with liver cirrhosis [1–4]. The prevalence of PVT increases with the severity of liver disease, being 1% in individuals with compensated cirrhosis and up to 8–25% in candidates for liver transplantation [1, 3–5].

In individuals with cirrhosis, reduced blood flow velocity in the portal vein seems to be the most important local factor responsible for the development of PVT [3, 6]. Several clinical risk factors have been shown to be associated with PVT: they include thrombocytopenia, previous variceal hemorrhage, splenectomy, surgical portosystemic shunt, and endoscopic treatment of esophageal varices [4, 7]. However, instead of being causative, these factors are probably a reflection of the severity of portal hypertension, which is by itself an important risk factor for PVT [4]. More recently, the recognition of a procoagulant imbalance in individuals with advanced liver disease has also been put forward in explaining the development of PVT in this population [1–4, 8]. Indeed, it is now clear that individuals with cirrhosis have a decreased production of liver procoagulant factors (with the exception of factor VIII) and also a decreased production of anticoagulant factors. The resulting procoagulant imbalance can be demonstrated in particular through the partial resistance to the anticoagulant action of thrombomodulin (a potent activator of protein C). The resistance to thrombomodulin is probably related to the markedly increased plasma levels of factor VIII and the concomitant decrease in protein C levels seen in advanced liver disease [1–4, 8]. Although contradictory results have been reported, a defect in fibrinolysis due to decreased plasma levels of plasminogen and increased levels of plasminogen activator inhibitor could also contribute to the procoagulant imbalance found with cirrhosis [3].

The clinical impact of PVT on liver function is still a matter of great debate in the literature. PVT is a well-known risk factor of early mortality after liver transplantation and can also contraindicate liver transplantation in cases where thrombosis extends to the splenomesenteric confluence [1, 5, 7–9]. PVT is also a predictive factor for mortality, independent of MELD score, in individuals with cirrhosis: the relative risk of death having been shown to be around 2.5 [5, 8, 9]. Because PVT by itself also increases portal hypertension, it increases the risk of variceal bleeding and has been described to be an independent risk factor for the inability to control variceal bleeding [1, 8, 10]. PVT can also be a life-threatening emergency when thrombosis extends to the superior mesenteric vein in which case it may lead to intestinal infarction [1, 4, 11]. Finally, it has been demonstrated that primary prophylaxis of PVT with low-dose LMWH was effective in reducing mortality and the risk of hepatic decompensation in a cohort of moderately severe cirrhotic individuals (Child B7-C10) [12]. 

The optimal management of PVT in individuals with cirrhosis is currently not addressed in any consensus publication or practice guidelines [4, 13, 14]. In the present systematic review, we explore the different aspects of the management of PVT in individuals with cirrhosis (excluding cases associated with hepatocellular carcinoma).

## 2. The Benefits of PVT Anticoagulation in Cirrhotic Individuals

To date, only few studies have evaluated the benefits of anticoagulation in individuals with cirrhosis. An obvious goal of anticoagulation is PV recanalization: when cirrhotic individuals with PVT are treated with anticoagulation, complete recanalization has been described in 33–45% while partial PV recanalization is observed in 15–35% of cases [1, 7, 8]. These rates of recanalization are similar to what is described in cases where PVT occurs in noncirrhotic individuals [15]. 

Senzolo et al. have conducted the largest study published to date on that topic by prospectively enrolling 56 individuals (35 in the treatment group and 21 in the control group) [6]. In the treatment group, 31% had complete PVT and 69% had partial PVT. Thirty-three out of the 35 treated individuals received low molecular weight heparin (LMWH); 2 individuals did not receive anticoagulation because of cavernous transformation. Complete recanalization was achieved in 12/33 (36%) individuals and partial recanalization in 9/33 (27%) individuals, after a mean of 5.5 ± 2.6 months (1–10 months). In univariate analysis, previous bleeding caused by portal hypertension (RR 3.1; CI 1.3–6.9; *P* = 0.01), time between diagnosis and inclusion in the study <6 months (RR 3.5; CI 1.5–8.5; *P* < 0.001), and time between diagnosis and anticoagulation <6 months (RR 3.3; CI 1.2–9.4; *P* = 0.004) were positively associated with PV recanalization. This study also demonstrated that anticoagulation could prevent PVT progression. In the treatment group, 15% of the individuals had progression of their thrombosis compared to 71.4% in the control group (*P* < 0.001).

Another study conducted in Spain by Delgano et al. included 55 cirrhotic individuals with acute/subacute PVT or a progressive splenomesenteric thrombosis [1]. The mean MELD score was 12.8 and thrombosis was partial in 75% of the individuals. In this study, 29 individuals (53%) were treated with vitamin K antagonists (VKA) and 26 individuals (47%) received LMWH. Therapy was administered for a median of 6.3 months and individuals were followed for a median time of 19 months. Complete PV recanalization was achieved in 45% of the cases and partial recanalization in 15% of the individuals. The only predictive factor for complete PV recanalization in this study was early initiation of anticoagulation after diagnosis (<14 days).

Another important study was conducted by Francoz et al. in 2005 [7]. This case-control study included 29 cirrhotic individuals with PVT on the waiting list for liver transplantation. PVT was partial in 20 individuals (69%) and complete in 9 individuals (31%). Ten individuals (between 1996 and 1998) did not receive anticoagulation therapy and were compared to 19 treated individuals (between 1999 and 2001) who received VKA therapy. In the 10 individuals not receiving therapy, PVT remained stable in 4 individuals and progressed in the other 6. In the 19 treated individuals, complete PV recanalization was achieved in 8 individuals (42%). The difference was statistically significant and in favor of anticoagulation therapy. In this study, there was no evidence that anticoagulation therapy increases blood loss during liver transplantation or that it increases the duration of surgery. 

Finally, Amitrano et al. published a study where 28 individuals with PVT were treated with LMWH [11]. PVT was partial in 83% of the cases and 46% of individuals had CHILD B or C cirrhosis. All individuals received enoxaparin 200 U/kg/d for 6 months. After 6 months, the patency of the PV was evaluated and therapy was continued if partial response was demonstrated and was discontinued if complete response or no response to treatment was observed. At 6 months, complete PV recanalization was achieved in 33% of the individuals while partial PV recanalization was achieved in 50%. In individuals with partial response to therapy, complete PV recanalization was achieved in 86% with the continuation of enoxaparin for an extra 6 months. Globally, complete PV recanalization in this study was achieved in 75% of the individuals after a median of 6.5 months.

## 3. Selection of Individuals for Anticoagulation Therapy

Even if anticoagulation therapy is associated with good rates of PV recanalization, the indications for treating PVT in cirrhotic individuals are not well defined in the current guidelines and consensus publications [4, 14]. In fact, the impact of PVT on the evolution of cirrhosis is still a matter of great debate [11] and the clinical benefits of PV recanalization have been demonstrated in only few particular situations. 

To date, there is accumulating evidence that cirrhotic individuals with PVT on the waiting list for liver transplantation should be treated with anticoagulation therapy. Indeed, Francoz et al. have demonstrated that complete or partial PV recanalization was associated with a better 2-year survival rate after liver transplantation (82-83% in individuals with partial and complete PV recanalization and 50% in individuals with complete PVT) [7]. This observation is supported by 2 other studies [16, 17]. One study showed a 32% increase in mortality in individuals undergoing liver transplantation with PVT [16]. The other study showed that the negative impact of PVT on posttransplantation survival was restricted to individuals with an MELD score <15 at the time of surgery [17]. The increased mortality and morbidity associated with PVT are mostly restricted to the first year after liver transplantation [7, 11]. It has also been shown that individuals with PVT at the time of liver transplantation are at higher risk of recurrent PVT after transplant and of requiring retransplantation [8].

Two other situations seem to be logical indications for anticoagulation in cirrhotic individuals with PVT. In acute PVT with extension to the superior mesenteric vein, despite the absence of data, the benefits of anticoagulation seem to exceed the potential risks of intestinal infarction [4, 14]. It also seems reasonable to consider anticoagulation therapy for PVT in cirrhotic individuals with a well-characterized prothrombotic disorder (i.e., the presence of a JAK-2 mutation). In all the other situations, the benefits of anticoagulation are largely unknown.

## 4. Anticoagulation Regimens for PVT in Cirrhotic Individuals

The optimal anticoagulation regimen for the treatment of PVT has not been determined yet and no clear recommendations exist regarding this question in recent guidelines and consensus publications [4, 14]. The choice of anticoagulation regimen is particularly difficult in the cirrhotic individual, mostly because anticoagulation monitoring is complex in this particular situation. 

VKA have been used in some studies to treat PVT in cirrhotic individuals. The rates of complete PV recanalization in cirrhotic individuals treated with VKA are between 42% and 45% [1, 7]. In the study conducted by Francoz et al. in 29 individuals on the waiting list for liver transplantation, complete PV recanalization was achieved in 42% of cases after a mean of 8.1 months of anticoagulation therapy [7]. Of interest, the mean INR before the initiation of treatment was 1.7. In the largest study published on the subject, Delgado et al. treated 29 of the 55 included individuals (53%) with VKA [1]. Complete PV recanalization was achieved in 45% and partial recanalization in 15%. The mean and median INR before VKA therapy was 1.3 (1.1–1.57). In these 2 studies, the target INR was between 2 and 3, with attempt to get as close as possible to 2.5. 

The most problematic issue with the use of VKA in cirrhotic individuals is the INR monitoring under therapy. The problem arises from the fact that conventional INR seems to be unreliable in this particular situation [8]. INR has only been validated in individuals with normal liver function on stable anticoagulation [18]. A 29% variation in mean INR has been reported in cirrhotic individuals in a study when three different thromboplastin reagents were used [19]. It is also unclear if a target INR between 2 and 3 is adequate in individuals with abnormal INR values before anticoagulation therapy [3, 8, 11]. Some authors have also raised the potential risk of further lowering protein C levels with the use of VKA: this could theoretically increase the prothrombotic imbalance of individuals with cirrhosis [3, 8]. 

LMWH has also been used to treat PVT in cirrhotic individuals. In their study, Amitrano et al. included 28 individuals with PVT [11] who were all treated with enoxaparin: complete PV recanalization was achieved in 33% of cases after 6 months of treatment and in 75% of the cases when LMWH therapy was extended an extra 6 months (6–17 months, median 6.5 months). A second study of 38 individuals treated with LMWH reported complete PV recanalization of 50% at 6 months [24]. In a third study, Senzolo et al. reported a 36% complete recanalization rate with nadroparin after a mean of 5.5 months (1–10 months) [6]. LMWH has also been shown to lead to similar rates of portal vein recanalization in individuals with PVT but without cirrhosis [11].

Despite these favorable observations, LMWH therapy is not without any risk either. In the literature, there is little information on the pharmacodynamic profile of LMWH in cirrhotic individuals [20]. Another important issue is that LMWH dosage is based on weight [21]. Cirrhotic individuals often have an increased volume of distribution because of ascites and edema which makes it difficult to determine the optimal dose of LMWH [21]. Rescent articles also point to the fact that monitoring of anti-X_a_ cannot be used to guide therapy in cirrhotic individuals [8, 20, 21]. Anti-X_a_ activity is not a direct measurement of the functional anticoagulant effect of LMWH, but it is instead a surrogate for LMWH concentration in the blood. This measurement is dependent on antithrombin-III (AT) levels, which are decreased in cirrhotic patients [21]. The lower levels of AT found in cirrhosis cause a falsely decreased anti-X_a_ activity. Therefore, in the particular case of cirrhosis, anti-X_a_ activity is not reliable to evaluate the anticoagulatory effect of LMWH and should not be used to guide anticoagulation therapy because it could be associated with an increased risk of bleeding [21]. Finally, renal function is often altered in cirrhotic individuals (particularly those awaiting liver transplantation): it is well recognized that LMWH is eliminated by the kidneys and that their half-life is increased in that context.

To avoid all the aforementioned problems, an interesting solution could be the use of direct thrombin inhibitors [3]. The potential advantage of these new drugs is that their mechanism of action is independent of AT. However, to date, trials studying direct thrombin inhibitors have specifically excluded cirrhotic individuals.

The choice of the anticoagulation regimen also needs to take into account the potential need to reverse the effect of anticoagulation: this can become necessary in cases of acute bleeding and in all cases undergoing surgery. Whereas the effect of VKA can be quickly and effectively reverted though prothrombin complex concentrate, there is yet no potent and rapidly acting antidote to the effect of LMWH or thrombin inhibitors.

## 5. Duration of Anticoagulation 

The ideal length of anticoagulation therapy for PVT in cirrhotic individuals is not known. However, in the above mentioned studies, a trend for better recanalization rates seems to stand out with early initiation of therapy. In the study published by Delgano et al., the initiation of anticoagulation <14 days after the diagnosis of PVT was the only predictive factor for complete PV recanalization [1]. In the recently published study of Senzolo et al., early anticoagulation (<6 months after diagnosis) was associated with an odds ratio of 3.3 (CI 1.2–9.4, *P* = 0.004) for complete PV recanalization [6]. In this same study, no PV recanalization was observed if anticoagulation therapy was initiated more than 10 months following the diagnosis of PVT. Consequently, these studies strongly argue in favor of early initiation of anticoagulation therapy. One has however to concede that the diagnosis of PVT is often made fortuitously thus making it difficult to determine when the thrombus started to develop. 

No consensus exists also in the optimal duration of anticoagulation therapy in that setting. As shown in the study published by Amitrano et al., individuals with partial response to anticoagulation at 6 months of therapy might benefit from prolonged therapy up to 12 months [11]. In individuals with partial PV recanalization after 6 months, complete recanalization could be achieved in 86% of the cases after a median time of 11 months when anticoagulation was continued, (7–17 months) [11]. In the study conducted by Senzolo et al., it was also shown that continuation of anticoagulation after 12 months in nonresponders was associated with a decreased risk of thrombosis progression (5/12 versus 15/21, *P* < 0.001) [6]. Finally, the study published by Delgano et al. showed high rates of PVT recurrence after discontinuation of anticoagulation [1]. In this study, 38.5% (5/13) of the individuals with complete PV recanalization during the study period stopped anticoagulation and developed recurrent PVT after a median time of 1.3 months following discontinuation of therapy. These studies all suggest that prolongation of therapy should be considered, especially in situations where PV patency is important, namely, in candidates for liver transplantation. 

## 6. Complications of Anticoagulation

In noncirrhotic individuals undergoing anticoagulation for PVT, this therapy is considered safe [11, 22, 23]. Indeed, Condat et al. have shown that anticoagulation did not increase the risk (RR 0.9; *P* = 0.9) or the severity of bleeding given that individuals received adequate prophylaxis for gastrointestinal bleeding [22]. However, anticoagulation is more complex in the setting of cirrhosis mostly because of the inherent risk of bleeding secondary to portal hypertension, which can be life threatening [2, 20]. However, it is generally accepted that gastrointestinal bleeding associated with portal hypertension is highly dependent on portal pressure. Any underlying coagulopathy, be it secondary to the liver disease itself or to anticoagulation therapy, should not precipitate bleeding, but could certainly make the bleeding more severe [20]. Therefore, bleeding complications in individuals with cirrhosis undergoing anticoagulation therapy for PVT should not be more frequent.

In published studies, the incidence of bleeding complications has been <5%. In the study published by Francoz et al. where 19 individuals were treated with VKA for a mean time of 8.1 months, only one individual developed a bleeding episode due to postendoscopic variceal ligation ulcer in the esophagus [7]. This individual was successfully treated with proton pump inhibitor and received two packed red blood cells. However, in this study, no information was given on the severity of portal hypertension and if prophylaxis against gastrointestinal bleeding was administered or not. In the study by Amitrano et al., where 28 individuals received enoxaparin, two cases of anemia (hemoglobin drop of 1.5 and 2.0 g/dl, resp.) apparently caused by severe portal hypertensive gastropathy have been described [11]. No case of variceal bleed occurred. In this study, all individuals had screening for esophageal varices and prophylaxis was given to all individuals with varices. In the study published by Delgado et al., during the 19-month study period, 6 variceal bleed occurred but were considered as probably not related to the anticoagulation therapy [1]. However, 5 further bleeding episodes considered secondary to anticoagulation occurred: 1 lower gastrointestinal bleeding, 1 obscure gastrointestinal bleeding, 1 vaginal bleeding, 1 bleeding after dental extraction, and 1 surgical wound hemorrhage. A platelet count <50  ×10^9^/l was the only factor more frequently associated with bleeding. The use of VKA showed a trend toward increased risk of bleeding but did not reach statistical significance. In the most recently published study, Senzolo et al. showed that bleeding complications secondary to portal hypertension were, in fact, more frequent in cirrhotic individuals with PVT not administered anticoagulation therapy [6]. In that control group, 5 episodes of variceal bleed occurred whereas only one case occurred in the treated group (*P* = 0.09). One individual in the untreated arm died due to a variceal bleed. None of these bleeding complications were secondary to a postligation ulceration in the esophagus. However, in the group receiving anticoagulation therapy, 3 bleeding complications occurred that were not related to portal hypertension (1 epistaxis, 1 hematuria, and 1 cerebral hemorrhage). The individual with intra-cranial bleeding remained with permanent neurologic deficits and this individual had no other risk factor for severe bleeding (platelets count at 110 × 10^9^, normal INR and normal creatinine). In a different type of study published in 2008, cirrhotic individuals receiving anticoagulation therapy for deep vein thrombosis presented bleeding complications in 35% of the cases [24, 25]. In this study, the severity of portal hypertension was not addressed and the risk of bleeding risk was higher in individuals receiving VKA. 

## 7. Variceal Bleed Prophylaxis 

In the previously described studies, the rate of variceal bleed was low given that individuals had prophylaxis for gastrointestinal bleeding. Therefore, if anticoagulation for PVT in a cirrhotic individual is to be performed, it is preferable to screen for varices before starting anticoagulation. However, in this particular situation, there is no current consensus or guidelines on whether nonselective beta-blockers, endoscopic variceal ligation (EVL), or combination therapy is better for variceal bleed prophylaxis [2, 4, 13, 14]. In the study published by Senzolo et al., all individuals underwent endoscopic screening for varices at inclusion [6]. Individuals with previous variceal bleed, grade II esophageal varices with red signs or grade III varices were treated by EVL before anticoagulation. The mean number of EVL procedures required to achieve eradication of varices was 2 (1–3 sessions). Anticoagulation therapy was started 15 days after the last EVL. In this study, the authors give no information on the use of beta-blockers. No bleeding secondary to post-EVL ulceration and only one case of variceal bleed occurred under anticoagulation therapy. In Amitrano's study, the strategy used for the prevention of variceal bleeding was different [11]. In the 14/28 individuals presenting with variceal bleed at the time of PVT diagnosis, endoscopic EVL was performed until eradication before starting anticoagulation. The median time from diagnosis to the eradication of varices was 4 months. These individuals also received nonselective beta-blockers before and during anticoagulation therapy. The 14 individuals not presenting with variceal bleed underwent variceal screening and received nonselective beta-blockers if medium-large varices were discovered (no variceal banding). In this study, no case of variceal bleed or post-EVL ulceration was reported. In the Delgano's study, anticoagulation therapy was started after appropriate primary or secondary prophylaxis for variceal bleed. No specific information was given regarding the type of prophylaxis, but 78% of individuals were on nonselective beta-blockers at time zero. 

There is a small but definitive and uncontrollable risk of hemorrhage secondary to post-EVL ulceration. Because of this fear, in most studies, the beginning of anticoagulation for PVT has been delayed until complete eradication of varices. However, this delay, as already discussed, could be associated with a lower rate of PV recanalization [1, 6]. One study conducted by Jasmohan et al. in 2008 has looked at the risks of performing EVL at the same time as anticoagulation [26]. A cohort of 5 individuals with esophageal varices (4 with cirrhosis) underwent EVL while on anticoagulation therapy. All individuals had grade 3 or more varices and had therapeutic INR (mean INR 2.3) when ligations were performed. All individuals received non-selective beta-blockers. The mean number of EVL procedures was 3.2/individuals (1–5 sessions). No bleeding complication was reported during the two weeks following each EVL. This small observational cohort needs to be put in the context that post-EVL hemorrhage is thought to occur at a rate of 3–15% [27–29]. 

Therefore, at this time, no definitive recommendation can be made regarding the optimal prophylaxis against variceal bleed in cirrhotic individuals undergoing anticoagulation for PVT. One needs to determine the importance of starting early anticoagulation in order to achieve rapid portal vein recanalization in each individual versus the risk of bleeding associated with this approach. A careful strategy could be to use nonselective beta-blockers instead of endoscopic variceal ligation if medium-large varices that have not bled are discovered during screening. More studies are needed before recommendation can be made in favor of EVL under anticoagulation.

## 8. Conclusions and Future Directions

PVT is a common problem in cirrhosis, mostly in individuals with advanced liver disease. PVT is an important prognostic factor of cirrhosis and also bears significance in individuals undergoing liver transplantation. Anticoagulation therapy for PVT in cirrhotic individuals is associated with complete recanalization rates between 33% and 45% after 6 months. Prolonged anticoagulation could be associated with higher complete recanalization rates, lower rates of thrombosis extension, and lower rates of thrombosis recurrence after discontinuation of anticoagulation. To date, no recommendation can be made on whether VKA or LMWH should be preferably used in cirrhotic individuals with PVT. However, it would probably be safer to use LMWH in cirrhotic individuals with abnormal INR before the initiation of anticoagulation therapy. Of note, anti-X_a_ activity in cirrhosis should not be used to guide therapy with LMWH because of the reduced levels of AT. Bleeding complications secondary to portal hypertension in cirrhotic individuals undergoing anticoagulation for PVT seem to be low but prophylaxis for variceal bleeding probably needs to be administered to all patients. To date, no recommendation can be made on whether EVL, nonselective beta-blockers or combination therapy is better for prophylaxis. In this context, it seems relatively safe to refer to the AASLD guidelines for the management of esophageal varices in this particular situation. 

Finally, we cannot make any recommendation regarding the management of PVT in the setting of hepatocellular carcinoma (HCC). This condition needs to be looked for when one makes the diagnosis of PVT in a cirrhotic patient. It bears a different clinical significance and probably is determined by different pathogenic factors. Further studies are needed to determine the optimal management of this condition.
